# Expanding uncapped translation and emerging function of circular RNA in carcinomas and noncarcinomas

**DOI:** 10.1186/s12943-021-01484-7

**Published:** 2022-01-07

**Authors:** Yan Wang, Chunjie Wu, Yu Du, Zhongwei Li, Minle Li, Pingfu Hou, Zhigang Shen, Sufang Chu, Junnian Zheng, Jin Bai

**Affiliations:** 1 Cancer Institute, Xuzhou Medical University, 84 West Huaihai Road, Xuzhou, 221002 Jiangsu Province China; 2 Center of Clinical Oncology, the Affiliated Hospital of Xuzhou Medical University, Xuzhou, Jiangsu China; 3 Department of Pharmacy, the Affiliated Hospital of Xuzhou Medical University, Xuzhou, Jiangsu China; 4 Jiangsu Key Laboratory of New Drug Research and Clinical Pharmacy, Xuzhou Medical University, Xuzhou, Jiangsu China

**Keywords:** Translation, Cap-independent, Circular RNA, Protein, Carcinomas

## Abstract

Circular RNAs (circRNAs) are classified as noncoding RNAs because they are devoid of a 5’ end cap and a 3’ end poly (A) tail necessary for cap-dependent translation. However, increasing numbers of translated circRNAs identified through high-throughput RNA sequencing overlapping with polysome profiling indicate that this rule is being broken. CircRNAs can be translated in cap-independent mechanism, including IRES (internal ribosome entry site)-initiated pattern, MIRES (m^6^A internal ribosome entry site) -initiated patterns, and rolling translation mechanism (RCA). CircRNA-encoded proteins harbour diverse functions similar to or different from host proteins. In addition, they are linked to the modulation of human disease including carcinomas and noncarcinomas. CircRNA-related translatomics and proteomics have attracted increasing attention. This review discusses the progress and exclusive characteristics of circRNA translation and highlights the latest mechanisms and regulation of circRNA translatomics. Furthermore, we summarize the extensive functions and mechanisms of circRNA-derived proteins in human diseases, which contribute to a better understanding of intricate noncanonical circRNA translatomics and proteomics and their therapeutic potential in human diseases.

## Introduction

Protein-coding genes annotated in the Human Genome Project account for only approximately 2.5% of the human genome [1]. The remaining transcripts in the genome are considered noncoding RNAs (ncRNAs) comprising circular RNAs (circRNAs), microRNAs, long noncoding RNAs, PIWI-interacting RNAs, and others [2]. CircRNAs are a unique subclass of ncRNAs characterized by covalently closed loops without 5’ and 3’ terminals [3, 4]. Generated from back splicing (head-to-tail splicing) events or noncolinear splicing reactions of precursor mRNAs (pre-mRNAs) in the nuclear and mitochondrial genomes of mammals [5–14], circRNAs have exclusive signs termed “back splicing junctions (BSJs)” to distinguish them from cognate linear RNAs.

Sanger HL and coauthors first discovered peculiar single-stranded covalently closed molecules in viroids by utilizing electron microscopy and created the term “circular RNA” [15]. Subsequently, these strange molecules were observed in pathogens and eukaryotic cells [16], but they have received little attention since for a long time they were considered to be functionless products of exon aberrant splicing. In 2013, a breakthrough in two articles published in NATURE revealed the sponge-like function of circRNAs in microRNAs [6, 17], transforming them from “waster to treasure (research hotspots)”. Recently, vast numbers of functional circRNAs were discovered in the eukaryotic tree of life from fungi to mammals, conserved across varied species [18–21]. These molecules are enriched in specific tissues/or cells or particularly developmental stages owing to their abundance or longer half-life [21–24]. The circular structure provides circRNAs with resistance against attacks by most ribonucleases except RNases encompassing RNase A, RNase T1, and RNase T2; as a result, circRNAs are more stable than their linear counterparts [21, 25, 26].

**Fig. 1:**
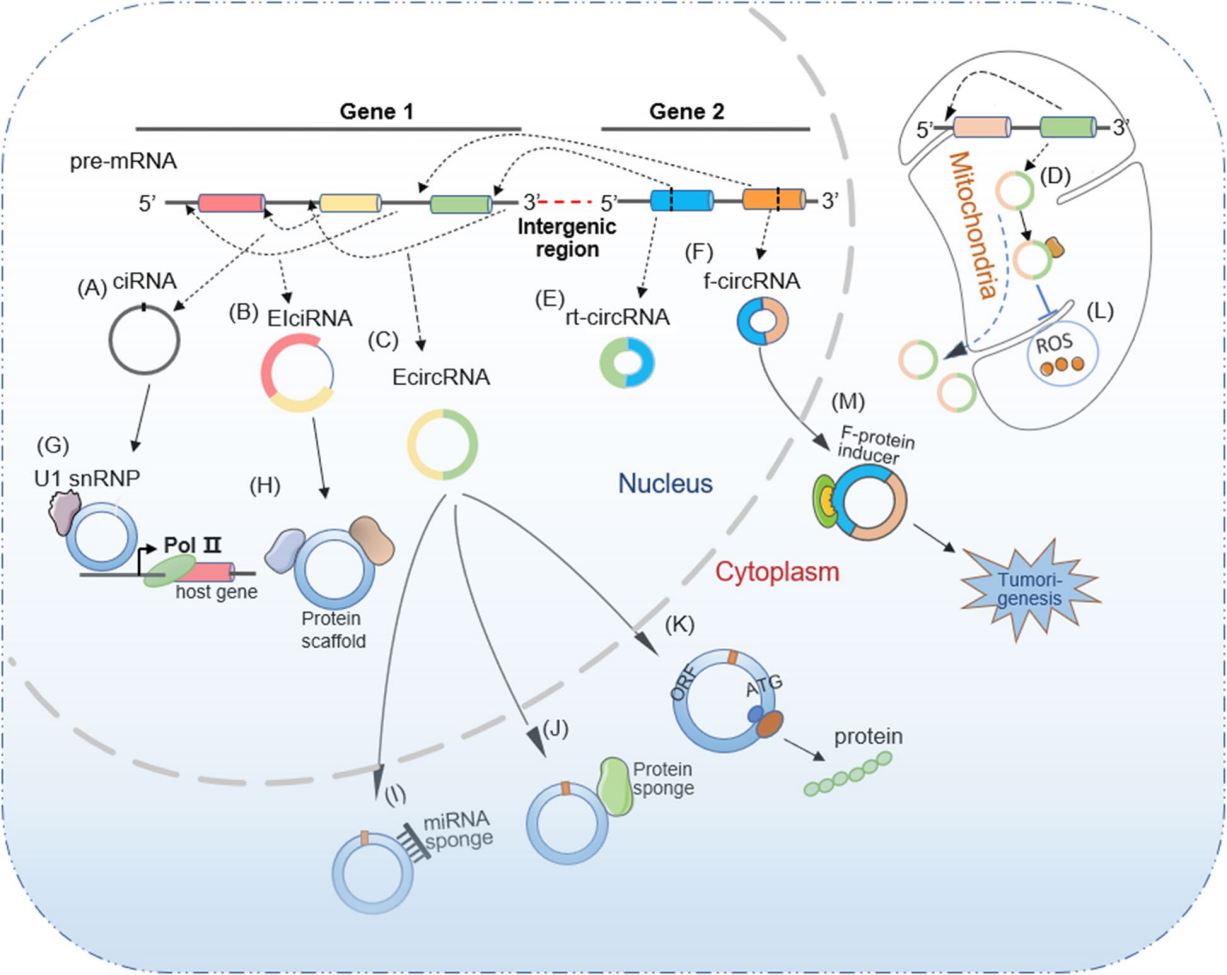
Biogenesis and function of various circular RNAs. Circular RNAs are the product of back splicing of pre-messenger RNAs (pre-mRNAs), comprising intronic circRNA (ciRNA) from intron (**A**), Exonic and intronic circRNA (EIciRNA) from exon covering intronic regions (**B**), exon circRNA (EcircRNA) from exons in nuclear (**C**) and mitochondrial genomes (mecciRNAs) (**D**), read-through circRNA (rt-CircRNA) from exons between neighboring genes on the same strand (**E**), and fused circRNA (f-circRNA) fuse from exons between two distant genes (**F**). CiRNA interacts with small nuclear ribonucleoprotein (snRNP) to enhance transcription rate of its host gene (**G**). EIciRNA can be a scaffold to recruit functional molecules (**H**). EcircRNAs are exported to cytoplasm as the sponges of micro-RNA (**I**) and protein (**J**), or as the templates to be translated into novel protein (**K**). MecciRNA may be linked to an inhibition of Reactive oxygen species-ROS (**L**). F-circRNA accompanied with fused protein to promote tumorigenesis (**M**)

Circular RNA classification is based on various origination and circularization modes and consists of intronic circRNAs (ciRNAs) circularized from introns (Fig. 1A), exon-intron circRNAs (EIciRNAs) from exons covering intronic regions (Fig. 1B), exon circRNAs (EcircRNAs) formed from mono- or multi-exons in nuclear genomes and mitochondrial genomes (mecciRNAs), certain exonic mitochondrial circRNAs may contain intron retained sequences (Fig. 1C-D) [8, 11–14, 27–29], read-through circRNA (rt-circRNA) from exons between neighbouring genes on the same strand (intrachromosomal chimaeras) (Fig. 1E), and fused circRNAs (f-circRNAs) that are fused exons between two distant genes (interchromosomal chimaeras) in the translocation process (Fig. 1F) [30–32]. Intriguingly, EIciRNAs and ciRNAs are sequestered in the nucleus [10, 29], while most EcircRNAs derived from the nuclear genome are exported to the cytoplasm [27, 30], and f-circRNAs may be localized in the nucleus and cytoplasm. The resultant discrepancy in localization is closely linked to their diverse functions. Nuclear ciRNAs and EIciRNAs are capable of enhancing the Pol II transcription rate of its host gene via interaction with the U1 small nuclear ribonucleoprotein (snRNP), as exemplified by circEIF3J and circPAIP2 (Fig. 1G), or as scaffolds to recruit functional molecules (circ-Amotl1, circ-Foxo3) (Fig. 1H) [7, 33–35]. F-circRNA derived from cancer-associated chromosomal translocations can be an oncogenic molecule involved in tumorigenesis (Fig. 1M) [31]. MecciRNAs are linked to the regulation of ROS in mitochondria (Fig. 1L) [12, 30–32], and the read-through circRNA function remains unclear [31]. Mature exonic circRNAs are exported to the cytoplasm as sponges of microRNAs and proteins (Fig. 1I-J) or as templates to be translated into novel proteins/peptides (Fig. 1K) [17, 36–39].

**Fig. 2:**
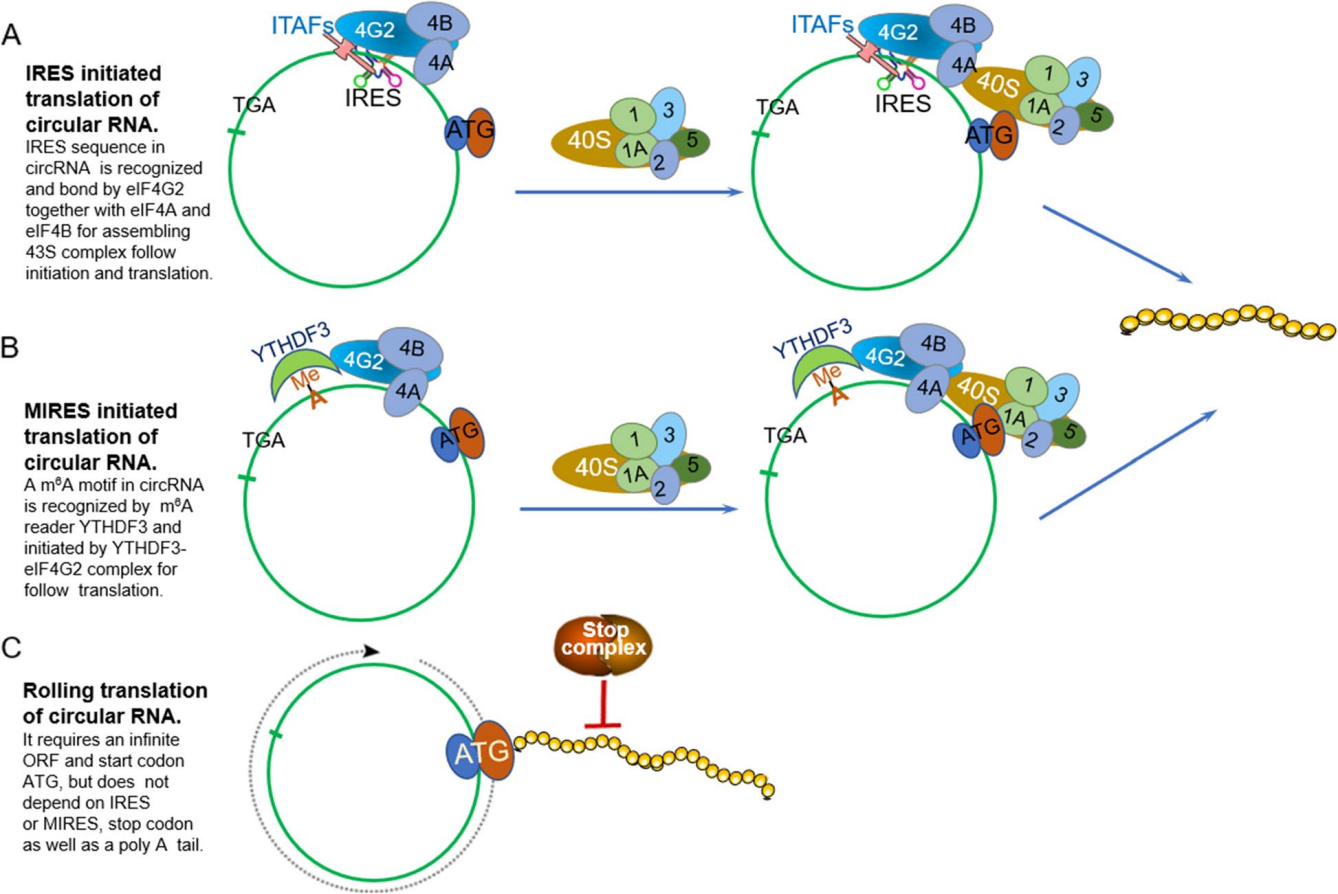
Translation mechanism of circular RNA. **A** IRES initiated translation of circular RNA. IRES is recognized and bond by eIF4G2 and acted as scaffold together with eIF4A and eIF4B for assembling 43S initiation complex including 40S ribosomal subunit and combination of eIF4G2 and eIFs complex to encounter start codon ATG for translation initiation and synthesis, which may be assisted with ITAFs (IRES trans-acting factors). **B** MIRES initiated translation of circular RNA. The m^6^A motif in circRNA is recognized by a m^6^A reader YTH domain family protein 3 (YTHDF3) to recruit eIF4G2 together with eIF4A and eIF4B for assembling 43S initiation complex including 40S ribosomal subunit and combination of eIF4G2 and eIFs complex to encounter ATG for translation initiation and synthesis. **C** circRNA harboring an infinite ORF and start codon ATG enables continuous translation termed rolling circle amplification without IRES element and stop codon, which can be terminated by a complex system named “programmed-1 ribosomal frameshifting”(-1PRF)-mediated out of-frame stop codon (OSC)

**Fig. 3:**
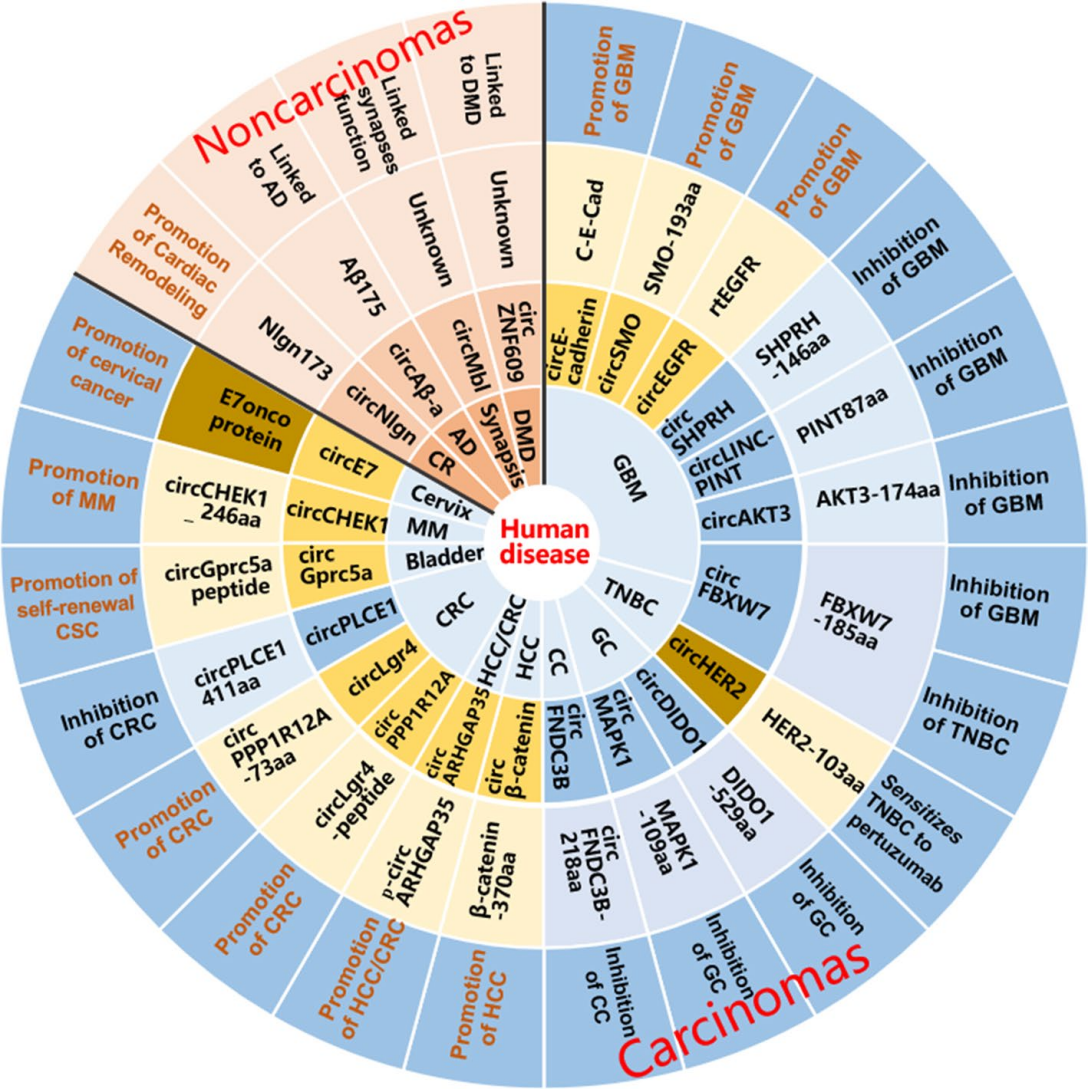
Translated circular RNAs and encoded proteins with various functions in carcinomas and noncarcinomas. Identified translated circular RNAs and encoded proteins with various functions in carcinomas containing glioblastoma (GBM), Triple-Negative Breast Cancer (TNBC), gastric cancer (GC), colon cancer (CC), colorectal cancer (CRC), hepatocellular carcinoma (HCC), multiple myeloma (MM), bladder cancer(Bladder), cervical cancer (Cervix), and in noncarcinomas diseases including Duchenne Muscular Dystrophy (DMD), synapsis function (Synapsis), Alzheimer’ s Disease (AD), cardiac remodeling (CR)

Previously, the translation of circRNAs was under speculation because canonical theory indicated that protein synthesis of eukaryotic mRNA in ribosome scanning mechanism requires the 5’ m^7^GpppN (m^7^G) cap and 3’ poly-A tail [40–43]. Due to the absence of the 5’ end and 3’ end for landing the m^7^G cap and poly-A tail, respectively, circRNAs were presumed to be untranslated. Moreover, traditional annotation of protein-coding genes only covers proteins more than 100 amino acids; thus, those shorter than 100 amino acids translated from circRNAs were ignored [44]. Recently, numerous translated circRNAs have been detected with the advent of advanced high-throughput technology, including RNA sequencing (RNA-seq) combined with polysome profiling and circRNA-specific bioinformatics algorithms [38, 39, 45, 46]. Natural circRNA translation is independent of the m^7^G cap but is dependent on internal ribosome entry (IRES) elements, including IRES or short IRES-like A/U enriched sequences [38, 45, 47–54], MIRES (m^6^A-IRES) [55, 56]. Moreover, circRNA can be translated in rolling circle amplification (RCA) mechanism (Fig. 2) [46, 57]. CircRNA-translated proteins (circ-proteins) modulate various physiopathologic processes ranging from carcinomas, including glioblastoma, colorectal and colon cancers, gastric cancer, hepatocellular carcinoma, and multiple myeloma [47, 56–62], and noncarcinomas, such as cardiac remodelling and Alzheimer’s disease (Fig. 3, Table 1) [66, 67].

**Table 1:** Overview of translated circRNAs and circ-proteins in carcinomas and noncarcinomas

Diseases	Translated circular RNAs				Circ-proteins						Ref.
	CircRNAs	Length (nt)	Ribosome binding	Translation mechanism	Circ-proteins	Length (aa)	Cellular location	Sequences covering domain of host protein	Expression (Up/Down)	Function and mechanism	
**Carcinomas**											
GBM	circE-cadherin (hsa_circ _0039992)	733	reads across BSJ in ribosome-Seq	IRES	C-E-Cad	254	Membrane, same to E-cadherin	Unique 14-aa tail at C-termini of C-E-Cad besides shared aa 282-522 at N-terminal of E-cadherin	Up	Unique 14-aa had new function that bond to the CR2 domain of EGFR, promoted STAT3 phosphorylation and nuclear translocation to interact with EGFRvIII, inducing GBM.	[46]
GBM	circSMO (hsa_circ _0001742)	727	In M and L polysomes fractions	IRES	SMO-193a.a	193	Cytoplasm and Membrane, same to SMO	Shared aa 230-421 at N-terminal of SMO covering most seven transmembrane domains besides one Glu at its C-termini	Up	Shared motif bond to N-terminal of SMO to translocate cholesterol, freed SMO from patched transmembrane receptors to maintain CSC self-renewal, inducing GBM.	[51]
GBM	circEGFR (hsa_circ_0080229)	249	In M and L polysomes fractions	Rolling translation	rtEGFR	83	Membrane, same to EGFR	shared aa 561-627 across extracellular domain IV of EGFR besides unique 19 amino acids, has similar function with EGFR	Up	Shared motif bond to extracellular Domain IV of EGFR to increase EGFR stability and membrane localization, attenuating its endocytosis and degradation, inducing GBM.	[57]
GBM	circSHPRH (hsa_circ_0001649)	440	undetected	IRES	SHPRH-146aa	146	unknown	shared aa1520–1651 covering the SNF2 domain of SHPRH besides 8-aa at its C-termini	Up	Shared motif as a decoy competitively bond to DTL to prevent its ubiquitination SHPRH which could ubiquitinate PCNA, inhibiting GBM.	[52]
GBM	circLINC-PINT (hsa_circ_0082389)	1084	two readers across BSJ by RNC-seq	IRES	PINT87aa	87	Nucleus, same to PINT	shared amino acids at N-terminal of PINT besides 10-aa at its C-termini	Down	Bond to the domain of PAF1 as an anchor keeping PAF1 complex on target genes’ promoter to repress transcriptional elongation and GBM.	[63]
GBM	circAKT3 (hsa_circ_0017250)	524	undetected	IRES	AKT3-174aa	174	Cytoplasm, similar to AKT3	shared aa 62-232 covering PH-domain and thr-308 of AKT3 besides 3-aa at its C-termini	Down	Shared motif bond to PDK1 to activate it, blocking AKT phosphorylation, inhibiting GBM.	[54]
GBM	circFBXW7 (hsa_circ_022705)	620	undetected	IRES	FBXW7-185aa	185	unknown	shared 167-aa with FBXW7a besides 18-aa at its C-termini	Down	Shared motif as decoy competitively bond to USP28, releasing FBXW7a to degrade c-Myc, inhibiting GBM.	[53]
TNBC		620	undetected	IRES	FBXW7-185aa	185	unknown	shared 167-aa with FBXW7a besides 18-aa at its C-termini	Down	Increased the abundance of FBXW7 and inducing c-Myc degradation, inhibiting TNBC.	[53]
TNBC	circHER2 (hsa_circ_0007766)	676	In M and L polysomes fractions	IRES	HER2-103	103	Membrane, same to HER2	shared 103 amino acids at N-terminal (aa 198-300) of HER2 covering most CR I domain of HER2 for EGFR/HER3 homo/heterodimer.	Up	Bond to CR I domain of HER2, stimulated EGFR/HER3 homo/heterodimer formation, phosphorylation and activation of AKT to sensitize Pertuzumab treatment of HER-103^**+**^ TNBC.	[64]
GC	circDIDO1 (hsa_circ_0061137)	1787	undetected	IRES	DIDO1-529aa	529	Nucleus, different from DIDO1-1a	shared 529 amino acids with NLS and PHD domain, lack of nuclear export sequence of DIDO1-1a	Down	Contrary to DIDO1-1a. bond to DNA binding domain and catalytic domain of PARP1 to block its activity, repressing GC,	[60]
GC	circMAPK1 (hsa_circ_0004872)	490	undetected	IRES	MAPK1-109aa	109	Cytoplasm, similar to MAPK1	shared aa 98-203 covering phosphorylated sites of MAPK1 besides 3-aa at its C-termini	Down	Contrary to MAPK1, competitively bond to MEK1 to block extracellular signals to intracellular signals for MAPK phosphorylation, repressing GC.	[59]
CC	circFNDC3B (hsa_circ_0006156)	526	undetected	IRES	circFNDC3B-218aa	218	Cytoplasm, similar to FNDC3B	shared 509 amino acids with FNDC3B besides 17-aa at its C-termini	Down	Attenuated Snail expression, enhanced FBP1-induced OXPHOS to repress CC.	[58]
HCC	circβ-catenin (hsa_circ_0004194)	1129	undetected	IRES	β-catenin-370aa	370	Cytoplasm, similar to β-catenin	shared 361 amino acids at N-terminal of β-catenin besides 9-aa at its C-termini	Up	As a decoy binding to GSK3β to prevent it degrade β-catenin, freed β-catenin activated Wnt/β-catenin pathway, promoting HCC.	[48]
HCC and CRC	circARHGAP35 (hsa_circ_0109744)	3867	in M polysomes fractions	m^6^A modification	p-circARHGAP35	1289	Nucleus, opposite to ARHGAP35	shared most amino acids at N-terminal of ARHGAP35 containing four FF domains lack of Rho GAP domain	Up	Contrary to ARHGAP35, shared motif interacted with nuclear TFII-I protein, promoting progression of HCC and CRC.	[56]
CRC	circPPP1R12A (has_circ_0000423)	1138	undetected	undetected	circPPP1R12A-73aa	73	unknown	shared 55 amino acids besides 17-aa unique tail	Up	Activated Hippo-YAP pathway to promote CRC, which is different from PPP1R12A.	[49]
CRC	circLgr4 (hsa_circ_02276)	unknown	undetected	undetected	circLgr4-peptide	19	Cytoplasm and nucleus	has 19-aa	Up	Interacted with extracellular domain LGR4 to activate Wnt/β-catenin pathway, promoting self-renewal and metastasis of CSC, driving CRC.	[50]
CRC	circPLCE1 (hsa_circ_0019223)	1570	undetected	IRES	circPLCE1 411	411	Cytoplasm	shared aa 1-403 at N-terminal of PLCE1 protein with distinct function with PLCE1, besides own 8-aa tail	Down	Bond to ATP binding domain of HSP90α to accelerate RPS3 to dissociate from the HSP90α/RPS3 complex, leading to the HSP70-induced ubiquitin-dependent degradation of RPS3 and suppression of NF-κB pathway, blocking CRC.	[61]
Bladder cancer	circGprc5a (hsa_circ_02838)	unknown	undetected	undetected	circGprc5a-peptide	11	unknown	unknown	Up	bind to Gprc5a to activate the GPCR signalling pathway and promote self-renewal and metastasis of cancer stem cells	[47]
MM	circCHEK1 hsa_circ_0024792	738	undetected	IRES	circCHEK1_246aa	246	unknown	shared N-terminus of CHEK1, has same function with CHEK1	Up	induced Chromosomal Instability and bone lesion formation by interaction with and decrease mutant CEP170	[62]
Cervical cancer	circE7	472	in polysomes fractions	m^6^A modification	E7 oncoprotein	98	Cytoplasm	Shared most amino acids with E7 protein harbouring unique sequences	Up	E7 oncoprotein is independent for the transforming activity of circE7 promoting progress of cervical cancer	[65]
**Noncarcinomas**											
CR	circNlgn (hsa_circ_0003046)	813	In heavy polysomes fractions	IRES	Nlgn173	173	Nucleus, different from Nlgn	9-aa tail at Nlgn173 C-termini for nuclear localization besides 164-aa at N- terminal of Nlgn	Up	Unique 9-aa motif interacts with the LaminB1 forcing nuclear localization of Nlgn173 to promote SGK3 and inhibit ING4, inducing Cardiac Remodelling, which is different Nlgn.	[66]
AD	circAβ-a (hsa_circ_0007556)	524	undetected	IRES-like A/U-rich sequences	Aβ175	175	unknown	remaining 158-aa approaching to C-terminal of Amyloid β peptide	Up	Its expression raised in brain tissues of AD patients	[67]
Synaptic function	circMbl	unknown	Ribosome binding	IRES-like UTR	none	unknown	Cytoplasm	unknown	Up	maybe linked to regulation of synaptic function	[45]
DMD	circZNF609	unknown	M and L polysomes	IRES-like UTR	none	753	unknown	unknown	Up	Linked to myoblast proliferation	[38]
Lifespan extending	circSfl	unknown	Ribosome binding	unknown	none	unknown	unknown	Sharing N-terminus with cytoplasmic and transmembrane domain	Up	extending lifespan of fruit flies	[68]

*ORF* Open Reading Framework, *BSJ* back splice junction, *IRES* Internal Ribosome Entry Site, *GBM* glioblastoma, *rtEGFR* rolling translation EGFR, *TNBC* Triple-Negative Breast Cancer, *GC* gastric cancer, *CC* colon cancer, *CRC* colorectal carcinoma, *HCC* hepatocellular carcinoma, *MM* multiple myeloma, *CR* Cardiac Remodeling, *AD* Alzheimer’s Disease, *DMD* Duchenne Muscular Dystrophy, *m*^*6*^*A*, N6-methyladenosine, *UTR* Untranslated Region

The current work summarizes the progress and exclusive characteristics of circRNA translation, highlighting the latest regulation mechanisms of circRNA translation and extensive function of circRNA-encoded proteins in human disease, which contributes to a better understanding of noncanonical circRNA-oriented translatomics and related therapeutic potential in human disease.

## Profiling of circRNA translation

### History of circRNA translation

In 1986, Wang and coauthors observed that a hepatitis delta viral genome-derived single-stranded circular RNA harboured an open reading framework (ORF), start codon AUG and stop codon, enabling translation of a polypeptide with 215 amino acids, indicating the potential of translation [69]. Similar translated circular RNAs were subsequently identified in plant viroids, virusoids, and the Sry gene of adult mouse testes [70, 71]. Such pioneering work failed to be accepted at that time because canonical theory assumed that protein translation of eukaryotic mRNA requires the m^7^G cap and poly A tail, both of which are absent in circRNAs [40, 42]. In 1995, one decade later, Chen and Sarnow showed an exception to this rule. They successfully created a circRNA carrying an ORF and IRES of encephalomyocarditis virus. This molecule generated an approximately 23 kDa protein product, suggesting that circRNAs can be translated in an IRES-dependent pattern independent of the m^7^G cap [72]. Nevertheless, they failed to demonstrate that natural circRNAs can be translated.

This challenge was overcome by AbouHaidar et al. in 2014. These scholars revealed that the covalently closed RNA from a virusoid produced a 16 kDa protein in an uncapped IRES-dependent mechanism [73]. Intriguingly, this translation used overlapping initiation-termination codons (UGAUGA). They speculated that overlapping codons may be generated by overlapping reading frames registered in the genome where the UGAUGA sequence becomes two consecutive opal termination UGA codons, causing overlapping start and stop codons [73]. In 2015, Wang et al. utilized minigenes containing GFP fragments and IRES sequences and directly demonstrated that in human and Drosophila cells, natural exonic circRNAs with putative ORFs could be translated into functional proteins in uncapped IRES-dependent pathways [74].

Abe et al. discovered an unexpected protein synthesis mechanism termed “rolling circle amplification (RCA)”. They created an exonic circRNA bearing an infinite ORF (iORF) and a start codon AUG and introduced it into rabbit reticulocyte lysate or human living cells, producing the expected protein bands despite the lack of a given IRES element, 5’m^7^G cap structure and a 3’poly-A tail [75, 76]. These findings support that circRNA can be translated in a cap-independent pathway with or without IRES, even the 3’polyA tail. However, circRNA translation remains debatable due to a lack of evidence of polysome involvement and any evidence of the biological functions of circ-proteins in human disease.

Through 2017, several exciting studies confirmed natural circRNA translation ability with proof of polysome involvement [38, 45, 55, 77]. Using ribosome footprinting (RFP) datasets, Pamudurti et al. identified 192 polysome-bound circRNAs (ribo-circRNAs) in rat/mouse tissues and 151 ribo-circRNAs in Drosophila. In particular, circMbl3 derived from the *muscleblind (mbl) gene* yielded a 37.03 kDa protein band detected by Western blot and MS analysis [45]. The IRES activity was determined by conducting a double luciferase assay and it was catalysed by overexpression of 4E-BP, an inhibitor of cap-dependent translation [45, 78]. Similarly, using a polysome sucrose gradient fractionation assay, Legnini et al. revealed that circ-ZNF609 was combined with heavy polysomes in human and mouse tissues. This ribo-circRNA generated an approximately 30 kDa protein in an IRES-like sequence (UTR)-directed mechanism [38].

Yang et al. discovered MIRES-dependent circRNA translation [55, 77]. They observed that numerous circRNAs harboured a higher density of m^6^A sites by m^6^A-RIP assay and an RRACH consensus motif of m^6^A modification approaching the start codon (R = purine, H = pyrimidine or A). One or two m^6^A sites in the circRNA were adequate to induce its translation [55]. Zhou et al. employed a genome-wide map of m^6^A circRNAs in human embryonic stem cells and observed hundreds of m^6^A methylation-modified circRNAs [77], implying the existence of MIRES-dependent circRNA translation. These data strongly confirm the capability of natural circRNA translation.

In 2018, Zhang and coauthors revealed that natural circRNA-translated proteins (SHPRH-146aa, FBXW7-185aa) perform inhibitory functions in the tumorigenesis of GBM [52, 53], defining the roles of circRNA-derived proteins (circ-proteins) in human disease. To date, dozens of functional circ-proteins have been found to be involved in various cancers and other diseases, which unveils just the beginning of the hidden translation omics of circRNA [46–54, 56–62, 66, 67].

### CircRNA translatomics characteristics

#### ORFs spanning back-splicing junctions

An open reading frame is a nucleic acid sequence starting with AUG (or CUG, GUG, ACG) and continuing in three-base sets for protein synthesis until it hits a stop codon [79]. The most significant difference in the ORF structure between mRNAs and circRNAs is that circRNA ORF requires a spanning back-splice junction with a minimum read-junction overlap of 9 nt on either side of the junction, and it recycles more than once [39]. The identified ORFs of circRNAs contain ORFs smaller than 100 aa, ORFs greater than 100 aa, and noncanonical infinite ORFs with start codons lacking stop codons (circE-cadherin, circEGFR) [46, 57, 75, 76], indicating the ORF complexity of circRNAs.

#### Start and stop codons in circRNA translation

Codons are nucleotide triplets within RNA encoding specific amino acids incorporated into a polypeptide chain, including the start codons and stop codons [80]. The start codon, consisting of a canonical AUG (ATG for RNA) or noncanonical start codons covering CUG, GUG and ACG, is the classic signal that tells ribosomes where to initiate eukaryotic mRNA translation [81, 82]. A stop codon refers to the signal to terminate polypeptide chain synthesis, involving one of three termination codons (UAG, UAA, or UGA) [80].

As the only start codon identified so far, ATG is indispensable for circRNA-related translation. However, stop codons are not necessary for rolling translation of circRNA except IRES/MIRES-dependent circRNA translation, which is distinct from mRNA-related translation [38, 39, 46, 57, 75, 76]. Intriguingly, overlapping initiation-termination codons (UGAUGA or “UAAUGA”) have been observed in certain translated circRNAs (circSHPRH, circPPP1R12A, and circ-AKT3) [49, 52, 54, 73], demonstrating a discrepancy between circRNA and mRNA translation.

#### Internal ribosome entry elements for circRNA translation

IRES is a crucial cis-acting RNA regulatory element that initiates factor complexes to recruit 40S ribosomal subunits for cap-independent protein translation [83]. Identified IRESs of circRNAs include typical IRES sequences or IRES-like sequences (A/U enriched sequences). Although circRNA IRESs harbour varied localizations between or behind the ORF or within the UTR of circRNA, these molecules exert the same activity to drive circRNA translation [38, 45, 67, 72, 84, 85]. MIRES, an m^6^A-modified structure (“RRACH”), is another internal element for initiating factor complexes to bind 40S ribosomal subunits to drive cap-independent protein translation [55]. MIRES in the 5’ UTR approaching to the start codon ATG of circRNA can be recognized by YTHDF3-eIF4G2 complex, which induces circRNA translation (Fig. 2) [86–89].

#### Ribosome-associated circRNA translation

Ribosomes are necessary for protein synthesis. They provide the synthesis environment, serve as a molecular scaffold to promote the interactions of codons contained in RNA and the anticodons in tRNA, and present peptidyl transferase activity, permitting the formation of peptide bonds between adjacent amino acids. Mono-/multiribosome-bound RNA is a signal for protein translation, which can be captured by advanced ribosome sequencing techniques [39, 90–92].

Ribosome-associated circRNAs can be predicted by ribosome profiling sequencing (Ribo-seq), ribosome footprint (RFP) dataset profiling, ribosome nascent-chain complex profiling sequencing (RNC-seq) and the Ribosome Atlas, or be detected by ribosome enrichment assays [38, 39, 46, 91]. To screen ribosome-bound circRNAs and diminish errors, filtering criteria in circRNA Ribo-seq require that more than 3 unique ribosome-binding reads and at least 5 total junction-spanning back-splice junction reads in circRNAs overlap to ensure the potential of ribo-circRNAs. Indeed, 40 ribosome-associated circRNAs in human heart tissues have been identified, pointing to the potential of circRNA translation [39]. The ribosome footprint refers to the 3-nt codon movement to manifest ribosome translation activity [90]. To select ribosome-combined circRNAs, the RFP assay requires that at least one RFPread covers the back-splice junctions of the circRNAs. Combined with the translating ribosome affinity purification (TRAP) assay, 37 ribo-circRNAs, including circ-ZNF-609, were found to be associated with light polysomes [45]. Independent of high concentrations of sucrose sediment and unconsolidated binding with ribosomes, RNC-seq is able to enrich more ribo-circRNAs; sometimes, it may result in biased analyses and higher false-positive rates. Sucrose gradient isolation of ribosome assays can be conducted to determine single ribosome-bound circRNA (ribo-circRNA) and their translation efficiency. Puromycin or EDTA treatment enables the evaluation of the activity of ribosomes during translation. The identified ribosome-associated circRNAs are illustrated in Table 1 [66].

### CircRNA translation mechanism and regulation

Protein synthesis in eukaryotes includes four phases: initiation, elongation, termination, and ribosome recycling [93, 94]. Translation initiation is the rate-limiting stage of translation. Canonical translation of eukaryotic mRNA depends on the m^7^G cap for recognition of the cap-binding protein initiation factor eIF4E complex, including eIF4E and eIF4G (a scaffold protein) and eIF4A (a helicase protein), and assembly of the 43S initiation complex to direct protein synthesis [42, 43]. In contrast, uncapped circRNA translation requires IRES or MIRES to combine with the initiation factor eIF4G2 or eIF3 complex containing eIF4G2, eIF4A and eIF4B, anchoring the 43S complex for protein translation (Fig. 2A-B) [72, 78], or it requires an infinite ORF and start codon to initiate translation in a rolling translation pathway (Fig. 2C) [46, 57, 75, 76], suggesting a discrepancy in the translation initiation pattern between cap-dependent and cap-independent translation.

### IRES-dependent circRNA translation

During IRES-dependent circRNA translation, IRES acts as the RNA scaffold to interact with alternative initiation factors eIF4G (eIF4G2 or DAP-5) or the eIF3 complex including eIF4A and eIF3, instead of the 5’cap-eIF4E complex to recruit 40S ribosomal subunits, followed by assembly of the 43S initiation complex to initiate translation (Fig. 2A) [72, 82, 95, 96]. Unexpectedly, certain lines of evidence revealed that the IRES activity of the virus could be regulated by IRES trans-acting factors (ITAFs), such as heterogeneous nuclear ribonucleoproteins (hnRNPs) [84, 97, 98]. hnRNPI can bind to two IRES activity sites within the EMCV IRES upstream of the AUG codon, stabilizing the IRES conformation for ribosome recruitment [99, 100]. hnRNPQ accelerates secondary structure unwinding of the IRES to enhance its activity and translation efficiency. PABPC1 and hnRNP U enable the recognition of noncanonical IRES-like elements (A/U-rich sequences) to promote cap-independent translation of circRNA [85]. QKI indicates two-faced effects on IRES activity as an inhibitor or promoter [36, 84]. HNRNPL elevates the back-splicing of circARHGAP35 by reorganizing CA-rich elements in flanking of its gene locus, resulting in an increase in oncogenic circ-protein (p-ARHGAP35) [56]. These data demonstrate the ability of ITAFs to control IRES activity and circRNAs biogenesis. The mechanism of these ITAFs in regulating IRES activity in endogenous circRNA translation remains unknown.

### MIRES-dependent circRNA translation

*N* ^6^-methyladenosine (m^6^A) is formed by methylated adenosine residues within RNA. m^6^A modification-triggered cap-independent translation mainly occurs in the 5’-UTR of certain circRNAs [55, 89]. CircRNAs bearing putative ORF spanning junctions and the m^6^A motif “RRACH” (R = G or A; H = A, C or U) in the 5’-UTR can be reorganized by YTH domain family protein 3 (YTHDF3, m^6^A reader), subsequently binding to the initiation factor eIF4G2 for anchoring and attachment of the 40S ribosomal subunit complex and the 43S complex to induce translation (Fig. 2B) [55, 87]. Interestingly, the eIF3-YTHDF1 or eIF3-METTL3 complex in the 3’UTR m^6^A sites and the single eIF3 in the 5’end of m^6^A sites have been found to be able to recruit the 40S ribosomal subunit for the translation of linear mRNA in response to stress [86–89]. The activity of these initiated complexes driving circRNA translation has not been determined.

### Rolling translation of circRNA

Given the unique rolling translation of circRNA, an infinite ORF and start codon (ATG) are sufficient for continuous translation, independent of IRES or MIRES elements [57, 75, 76, 78]. The RCA pattern is similar to an isothermal and enzymatic process induced by a particular group of DNA polymerases for the ultrasensitive detection of DNA. During this reaction, longer nucleic acids can be generated using an infinitely repeating circular template in a given period [101]. In certain translatable circRNAs, because of the looped structure, their nucleotides are not matched with integral multiples of three, and stop codons fail to be engaged in all reading frames or out of in-frame, causing the formulation of an infinite ORF [73], which offers the possibility of circRNA translation using the RCA mechanism. Furthermore, rolling circRNA translation is a considerably efficient and simpler process compared to canonical protein synthesis. The latter follows the ribosome scanning mechanism and requires complex recycling and reinitiation processes, attenuating the synthesis efficiency and protein outputs [42, 72].

It appears that artificial circRNA rolling translation is ceaseless due to an infinite ORF [75, 76]; in contrast, rolling translation in certain natural circRNAs can be terminated. Liu and coauthors discovered that with an infinite ORF and the start codon ATG, cirEGFR encoded a polymetric protein complex termed rtEGFR (rolling translation EGFR) in a rolling translation mechanism, while this process was terminated by a complex system named “programmed-1 ribosomal frameshifting” (-1PRF)- mediated out-of-frame stop codon (OSC) to break the endless movement of the codons (Fig. 2C) [57, 102, 103].

IRES- and m^6^A-mediated translation initiation and rolling translation are crucial mechanisms for circRNA translation (Fig. 2). Its regulatory mechanism in eukaryotic cells is still unclear.

## CircRNA-encoded proteins in carcinomas and noncarcinomas

### CircRNA-encoded proteins in carcinomas

#### 14-aa tail of C-E-Cad in response to GBM

A novel protein termed the E-cadherin protein variant (C-E-Cad) is translated from circE-cadherin (hsa_circ_0039992), and it is 254 amino acids in length. C-E-Cad harbours a unique 14 aa tail at the C-terminus due to a natural frameshift in the second-round translation of circE-cad, possessing a multiple-round ORF (Table 1) [46].

EGFR signal phosphorylation and activation are pivotal for the tumorigenicity of GBM [104]. C-E-Cad colocalized in the cell membrane with full-length EGFR and EGFRvIII, an active EGFR mutant frequently amplified and coexpressed with EGFR in GBM. The C-E-Cad 14-aa tail enables direct binding to the CR2 domain of full-length EGFR through salt bridge and hydrogen bond interactions, accompanied by EGFRvIII to promote STAT3 phosphorylation and nuclear translocation and AKT and ERK1/2 phosphorylation, resulting in the tumorigenicity of GBM [46, 105]. Consequently, C-E-Cad is an individual target for combined antibody treatment of glioblastoma because it facilitates malignant phenotypes, including proliferation, invasion, antiapoptosis, senescence resistance and cell stemness properties and sphere-forming frequency (Table 1).

#### SMO-193a.a, a scaffold for SMO cholesterol modification inducing GBM

A nascent protein with 193 amino acids termed SMO-193a.a. is generated from circSMO (hsa_circ_0001742). SMO-193a.a. shares the same 192 amino acids with the full-length SMO protein, which covers seven transmembrane domains responsible for cytoplasmic and membrane localization [51].

Hedgehog (HH) signalling is involved in the tumorigenesis of many cancers, including GBM, which is activated through the binding of HH to PTCH and derepressing SMO, releasing the Gli1 transcription factor for nuclear translocation and gene expression regulation [106, 107]. Cholesterol modification is indispensable for full-length SMO activation, which requires seven transmembrane domains for binding to cholesterol and PTCH1-blocked SMO cholesterol modification [107, 108]. SMO-193a.a. promotes cholesterol modification of full-length SMO by directly binding to N-terminus of SMO as a scaffold to translocate cholesterol to full-length SMO, functionally maintaining the cell stem cell (CSC) self-renewal ability and tumorigenicity of GBM [51].

#### rtEGFR, a platform enhancing EGFR stability to drive GBM

Rolling-translated EGFR (rtEGFR) is the polymetric protein complex translated from circEGFR (hsa_circ_0080229) in a rolling translation pattern due to an infinite ORF. Its core 83 amino acid sequence spans extracellular domain IV of the host protein EGFR, which comprises four crucial sites necessary for EGFR/rtEGFR interaction and EGFR activation. When colocalized in the cell membrane with full-length EGFR, rtEGFR can interact with extracellular domain IV of EGFR, maintaining EGFR stability and membrane localization, promoting the tumorigenicity of GBM [57]. Consequently, rtEGFR becomes an optimal target for monoclonal antibody or nimotuzumab-combined treatment of GBM to overcome nimotuzumab resistance [57, 109].

#### SHPRH-146aa, a decoy releasing SHPRH, blocking GBM

A newly discovered protein with 146 amino acids named SHPRH-146 aa is the product of circSHPRH (hsa_circ_0001649). It shares the same amino acids 1520-1651 at the C-terminus of full-length SHPRH containing two ubiquitination sites at K1562 and K1572 [52]. SHPRH is a well-characterized E3 ligase that targets proliferating cell nuclear antigen (PCNA) for degradation [110, 111]. Degradation induced by the E3 ligase DTL requires preferential interaction with the C-terminal sequence of SHPRH. Both SHPRH and SHPRH-146aa can be ubiquitin targets of DTL, while SHPRH-146aa possesses a stronger affinity. As a result, SHPRH-146aa acts as a decoy that competitively binds to DTL to release the host SHPRH from ubiquitination degradation. “Freed SHPRH” causes PCNA ubiquitination degradation to repress cell proliferation, reducing the tumorigenesis of GBM [52].

#### PINT87aa, a novel anchor for PAF1 to block GBM

A novel peptide named PINT87aa is translated from circLINC-PINT (hsa_circ_0082389), the unique circular form of a long intergenic noncoding RNA p53-induced transcript (LINC-PINT), mainly concentrated in the nucleus [63].

Although covering the same 77 amino acids as the N-terminus of the full-length PINT protein, PINT87aa exerts a unique tumour suppressive function that differs from that of the host PINT protein. It has been revealed that the PAF1 complex is involved in RNA II polymerase (Pol II) recruitment and regulation of the transcriptional elongation of downstream genes [112, 113]. PINT87 directly binds to the middle region (150-300 aa) of PAF1 and serves as an anchor, holding the PAF1 complex on the target gene promoter, hampering Pol II-induced mRNA elongation of multiple oncogenes (*CPEB1*, *SOX-2*) and abolishing cell cycle progression [63, 112–114].

#### AKT3-174aa, a blocker of PDK1-mediated AKT phosphorylation and GBM

A nascent protein with 174 amino acids termed AKT3-174 aa is generated from circAKT3 (hsa_circ_0017250). It covers the same amino acids 62-232 in the middle region of full-length AKT3 (PKBγ, protein kinase Bγ) containing a PH domain with the Thr308 site necessary for phosphorylation [54].

Notably, AKT3-174aa exerts an inhibitory function in GBM contrary to that of the host AKT protein [115]. PDK1-mediated AKT phosphorylation at Thr308 and activation are the initial key steps in activating the RTK/PI3K/AKT signalling pathway for GBM progression [116, 117]. Due to its higher binding affinity to phosphorylated PDK1 (p-PDK1), AKT3-174aa prefers to interact with activated PDK1, blocking Akt phosphorylation at Thr308 and negatively modulating PI3K/AKT signal intensity to reduce cell proliferation, resulting in the reduction of tumorigenicity and the radiation resistance of GBM [54].

#### FBXW7-185aa, a novel inhibiter of GBM and TNBC

FBXW7-185aa, a novel FBXW7a variant with 185 amino acids, is the product of circFBXW7 (hsa_circ_022705). It shares the same 165 amino acids at the N-terminus of the full-length FBXW7a protein, a crucial isoform of the E3 ligase FBXW7 [53]. The deubiquitinating enzyme USP28 reportedly binds to the N-terminus of FBXW7a for deubiquitination degradation and then induces c-Myc to promote GBM [118–120]. Since it possesses the same motif as FBXW7a but with a stronger affinity to USP28, FBXW7-185aa acts as a decoy and preferentially binds to USP28 to release FBXW7a. Freed FBXW7a induces c-Myc ubiquitin degradation, diminishing the cell proliferation and tumorigenesis of GBM [53].

Similarly, FBXW7-185aa demonstrates an inhibitory capability in triple-negative breast cancer owing to an enhancement of FBXW7 expression to induce c-Myc degradation (Table 1) [121].

#### HER2-103 sensitizes TNBC to pertuzumab

HER2-103, a new HER2 variant with 103 amino acids, is translated from circHER2 (hsa_circ_0007766). The sequence of these 103 amino acids is same as the CR I domain of full-length HER2, which is indispensable for EGFR/HER2-103 or HER3/HER2-103 dimerization and downstream signalling cascade activation [64, 122, 123] and it is localized close to the cell membrane, similar to the full-length HER2 protein. Accordingly, HER2-103 is able to promote TNBC cell proliferation and invasion by interacting with the CR I domain of HER2, stimulating EGFR/HER3 homo/heterodimer formation and phosphorylation, and sequential AKT activation, displaying obvious tumorigenicity [64]. Pertuzumab, an anti-HER2 antibody, is ineffective in TNBC patients lacking HER2 expression [124]. Notably, HER2-103-expressing TNBC cells and mouse xenografts are sensitive to pertuzumab treatment against certain TNBCs by acting on its shared CR I domain of HER2, which enables it to be an optimal target for anti-HER2 mono-antibodies such as pertuzumab or combined with trastuzumab [64, 124].

#### DIDO1-529aa, a new GC inhibitor

DIDO1-529aa translated from circDIDO1 (hsa_circ_0061137) is a new isoform of DIDO1-1a (death-inducer obliterator isoform 1-1a). It shares all 529 amino acids, similar to the DIDO1-1a protein, which contains NLS and PHD domains, while lacking the nuclear export sequence (NES) of the C-terminus, causing nuclear localization [60]. Interestingly, due to the absence of an NES, DIDO1-529aa demonstrates a new function independent of full-length DIDO1-1a; it inhibits GC through direct interaction of the DNA binding domain (DBD) and the catalytic domain (CAT) to block the activity of poly ADP-ribose polymerase 1 (PARP1). Moreover, it acts as a partner by binding to peroxiredoxin 2 (PRDX2) to induce an E3 ligase of the SCF ubiquitination complex for RBX1-mediated ubiquitination and degradation of PRDX2 and inactivation of its downstream signalling pathways, hampering the proliferation and invasion of GC [60, 125].

#### MAPK1-109aa, a novel blocker of MAPK1 signalling

A novel MAPK1 variant with 109 amino acids termed MAPK1-109 aa is encoded by circMAPK1 (hsa_circ_0004872). This variant shares the same sequences at amino acids 98-203 with the full-length MAPK1 protein, which includes indispensable MAPK1 phosphorylation sites [59]. MEK1 is a pivotal component of the Ras-Raf-MEK-MAPK cascade for the transmission of extracellular signals to intracellular signals and the phosphorylation of its downstream substrates, which is positively linked to cell growth and proliferation in numerous cancers [126, 127].

Interestingly, MAPK1-109aa exhibits the opposite function to full-length MAPK1. Due to sharing the same sequences, MAPK1-109a competitively binds to MEK1 to block the transmission of extracellular signals to intracellular signals to phosphorylate MAPK and downstream substrates encompassing p-c-Fos, p-c-JUN, and p-RSK, repressing the proliferation and invasion of GC cells [59].

#### CircFNDC3B-218aa, a novel CC inhibitor

CircFNDC3B-218aa is a novel protein with 218 amino acids translated from circFNDC3B (hsa_circ_0006156). It shares 201 amino acids with the N-terminal sequence of full length FNDC3B [58]. Tumorigenesis depends on an enhanced glycolytic phenotype switched from OXPHOS, termed the Warburg effect [128]. This metabolic shift can promote EMT progression, inducing tumour malignancy. The gluconeogenic enzyme fructose-1,6-bisphosphatase 1 (FBP1), one of the related-limiting enzymes in gluconeogenesis, exerts crucial function switching from glycolysis to oxidative phosphorylation (OXPHOS), which is necessary for tumour malignancy, blocking cancer progression via the Snail-FBP1 axis [129, 130]. CircFNDC3B-218aa presents an inhibitory ability to restrict tumorigenesis by attenuating Snail expression, enhancing FBP1-induced OXPHOS, as shown by the reduction of glucose uptake, pyruvate production and lactate production, and the promotion of metabolic reprogramming from glycolysis to oxidative phosphorylation [58].

#### β-catenin-370aa, a decoy of GSK3β releasing β-catenin to promote HCC

β-catenin-370aa, a nascent β-catenin isoform with 370 amino acids, is encoded by circβ-catenin (hsa_circ_0004194). It shares 361 amino acids at the N-terminus of the full-length β-catenin protein in addition to a 9-aa tail at the C-terminus. This novel molecule is found in the cytoplasm, unlike β-catenin, which is found in the nucleus [48]. As a vital component of the Wnt pathway, active β-catenin accumulates in the nucleus and participates in a variety of pathological events after being freed from glycogen synthase kinase 3β (GSK3β)-induced phosphorylation and proteasome-mediated ubiquitination degradation [131, 132]. Instead, β-catenin-370aa is localized in the cytoplasm and can be a decoy that preferentially binds to cytoplasmic GSK3β, protecting full-length β-catenin from GSK3β-directed degradation. Released β-catenin stimulates the Wnt/β-catenin pathway, promoting liver cancer progression [48].

#### p-circARHGAP35, an oncoprotein in HCC and CRC

P-circARHGAP35 is a novel oncoprotein with 1289 amino acids translated from circARHGAP35 (hsa_circ_0109744). This oncoprotein harbours the same amino acids with sequences at the N-terminus of the full-length ARHGAP35 protein, which encompasses four FF domains without the Rho GAP domain [56]. P-circARHGAP35 accumulates in the nucleus, unlike ARHGAP35 in the cytoplasm [133]. Accordingly, nuclear p-circARHGAP35 functions as an oncoprotein to facilitate tumour migration, invasion, and metastasis of cancer cells, including both hepatocellular carcinoma and colorectal cancer, owing to interaction with the transcriptional regulator TFII-I. This function is opposite to host ARHGAP35, a tumour inhibitor of RhoA activation in the cytoplasm [56, 133], demonstrating the complexity of the cancer transcriptome.

#### CircPPP1R12A-73aa and CircLgr4-peptide, novel CRC inducers

A novel protein with 73 amino acids named circPPP1R12A-73 aa is translated from circPPP1R12A (hsa_circ_0000423). It shares 56 amino acids with sequences at the N-terminus of full-length PPP1R12A. However, circPPP1R12A-73aa confers distinct functions to facilitate the proliferation and metastasis of CRC owing to its activation of the Hippo-YAP signalling pathway; nevertheless, full-length PPP1R12A does not show increased expression in CRC tissues [49].

The circLgr4 peptide is a small polypeptide with 19 amino acids encoded by circLgr4 (hsa_circ_02276) [50]. Lgr4, Lgr5 and Lgr6 are vital members of the Rspo/Lgr4/5 signalling pathway, which belongs to the bypass pathway of the Wnt/β-catenin signalling pathway [134]. CircLgr4 peptide function is closely dependent on its host protein Lgr4, one of the activated receptors in the noncanonical Wnt/β-catenin signalling pathway [135]. It interacts with LGR4 to efficiently activate Wnt/β-catenin signalling and promotes the self-renewal and metastasis of cancer stem cells [50].

#### CircPLCE1-411, a novel CRC inhibitor

CircPLCE1-411, a novel protein with 411 amino acids, is derived from circPLCE1 (hsa_circ_0019223). It shares the same amino acids 1-403 at the N-terminus of the full-length PLCE1 protein [61].

Ribosomal protein S3 (RPS3), a component of the 40S ribosomal subunit, harbours the ability to combine with the p65 subunit of NF-κB to drive nuclear translocation of the NF-κB complex [136]. HSP90α, a partner of RPS3, maintains RPS3 stability resistance to ubiquitin-dependent degradation [137, 138], while blocking the activation of the ATP-binding domain of HSP90 enables the release of RPS3 from the HSP90α/RPS3 complex [139, 140]. Freed RPS3 can preferentially bind and be degraded by the chaperone E3 ligase complex HSP70-CHIP [141]. Despite sharing the same sequences with the host PLCE1, circPLCE1-411 demonstrates a distinct capability to suppress colorectal cancer cell proliferation and migration via its interaction with the ATP-binding domain of HSP90α to accelerate the dissociation of RPS3 from the HSP90α/RPS3 complex, leading to HSP70-induced ubiquitin-dependent degradation of RPS3 and the suppression of NF-κB nuclear translocation and activation [61].

#### CircGprc5a-peptide, a bladder cancer promoter

CircGprc5a-peptide with 11 amino acids is the product of circGprc5a (hsa_circ_002838). G-protein-coupled receptor C family 5A (Gprc5a), a key membrane protein in the GPRC signalling pathway, is involved in the maintenance of self-renewal and metastasis of tumour stem cells [47, 142]. The function of circGprc5a-peptide is Gprc5a-dependent. It can drive bladder oncogenesis and metastasis accompanied by Gprc5a to activate the GPCR signalling pathway and promote the self-renewal and metastasis of cancer stem cells [47].

#### CircCHEK1_246aa induces MM

A nascent CHEK1 variant termed circCHEK1_246aa is translated from circCHEK1 (hsa_circ_0024792). Multiple myeloma (MM) cells expressed CircCHEK1_246aa is mainly in the CHEK1 kinase catalytic centre, and mature circCHEK1_246aa can be secreted into the bone marrow microenvironment. In this region it interacts with native centrosomal protein 170 (CEP170) to attenuate mutant *CEP170* expression in MM cells, promoting multiple myeloma (MM) by inducing chromosomal instability and bone lesion formation, exerting a similar function to full-length CHEK1 [62].

#### E7 oncoprotein transforms the activity of HPVs

The E7 oncoprotein, with 98 amino acids, is the product of circE7 originating from human papillomavirus 16. Its translation can be facilitated by QKI and heat shock stress. The ability of the E7 oncoprotein to suppress cervical cancer cell growth indicates that virus-derived circRNA translation may be responsible for the transforming properties of some HPVs [65].

### CircRNA-encoded proteins in noncarcinomas

#### Unique 9-aa of Nlgn173 confers cardiac remodelling

A nascent protein with 173 amino acids termed Nlgn173 translated from circNlgn (hsa_circ_0003046) harbours an exclusive 9 amino acid motif (GYRPAANWI) at the C-terminus responsible for nuclear localization, and the remaining 164 amino acids are the same as the full-length Nlgn protein [66].

Nlgn173 is highly expressed in hypertrophic and fibrotic hearts tissues, and its particular 9-amino-acid domain interacts with the structural protein LaminB1 to force Nlgn173 nuclear localization. Nuclear Nlgn173 combines with the helix-turn-helix (HTH) DNA binding motif at the promoters of glucocorticoid-inducible kinase-3 (SGK3) to induce cardiac fibroblast proliferation and inhibits growth protein 4 (ING4) to attenuate cardiomyocyte survival [66, 143, 144], finally leading to cardiac remodelling.

#### Aβ175 expression linked to AD

The novel polypeptide with 175 amino acids named Aβ175 is derived from circAβ-a (hsa_circ_0007556). This molecule shares the same 158 amino acids approaching the C-terminus of the full-length amyloid β peptide. Aβ175 expression is enhanced in brain tissues of Alzheimer’s disease patients, hinting at its potential to induce AD [66]. The detailed mechanism has not been investigated.

### Other functional peptides of circRNAs

Other peptides from translated circRNAs have been verified to be biologically functional. Pamudurti et al. certified that a nascent protein encoded from circMbl3 tends to be linked to the regulation of synaptic function [45]. CircZNF609-translated peptide is prone to regulate myoblast proliferation since circZNF609 is strongly expressed in human myoblasts with Duchenne muscular dystrophy (DMD) [38]. Another circ-protein from circSfl bears the same N-terminal sequence as full-length Sfl, including its cytoplasmic and transmembrane domains. Although the enzyme active domain is absent, this nascent protein is capable of extending the lifespan of fruit flies, playing an important role in regulating ageing in vivo [68].

## Conclusions and future perspectives

An accumulating number of circRNAs have been confirmed to be translated through uncapped translation mechanisms, including IRES- or MIRES-dependent pathways, o RCA mechanism (Fig. 2). The resultant proteins play various biological roles (Table 1). This noncanonical translatomics has attracted increasing attention, but several issues need to be addressed in the future.

First, the precise regulatory mechanism of natural circRNA translation remains unclear. ITAFs such as hnRNPI, hnRNPQ reportedly dominated the activity of certain viral IRESs [99, 100]. Additionally, Chen certified that a stem-loop structured RNA element in IRES termed SuRE was located 40-60 nt from the first nucleotide of the IRES of circular RNAs, particularly promoting translation of circRNAs instead of linear RNAs [145]. The detailed mechanism of ITAFs and SuRE involved in regulating IRES activity in natural circRNA translation in cells requires elucidation.

Second, engineering exosome-delivered translated circRNAs and their functions in human disease has yet to be defined. Rolling translation is a novel mechanism driving circRNA translation to efficiently generate a large number of protein products beyond that of cognate mRNAs [146], offering the possibility to utilize circRNA-derived proteins for future gene therapy [146, 147]. Scholars have revealed the potential of circRNA translation engineering by designing a novel expression system (rAAV) to induce the initiation of artificial circRNA translation [148] or natural circRNA translation in eukaryotic cells in order to increase the output and expression duration of circRNAs [146], whereas suitable vehicles delivering translated circRNAs into target organs are unknown. Exosomal circRNAs are stable regulators or biomarkers involved in human diseases [149, 150]. Exosome-transferred lncRNA RN7SL1 is able to stimulate CAR-T cells to accelerate autonomous and endogenous immune function [151]. Engineering of exosome-delivered translated circRNAs aimed at individual diagnoses and treatments appears to be a promising field.

Third, the development of more sensitive approaches is required to discover the low-abundance peptides from translated circular RNAs.

Finally, mature translated circRNAs are exported to the cytoplasm as templates for protein synthesis, while the resultant proteins exhibit various localizations and functions similar to or different from host proteins. How are translated circRNAs trafficked to the cytoplasm from the nucleus? Why do these proteins have different localizations and functions compared to their host proteins? These issues remain under-addressed thus far. Future work will be aimed at the elucidation of the regulatory mechanism underlying translation and function of circRNAs and the resultant proteins in pathophysiological processes, as well as the exploration of the therapeutic potential of translated circRNAs and exosome-coated translated circRNAs to benefit individual diagnoses and human disease treatments.

## Data Availability

Not applicable.

## Abbreviations

circRNA: Circular RNA
IRES: Internal ribosomal entry sequence
m^6^A: N6-methyladenosine
RCA: Rolling translation mechanism
ncRNAs: Non-coding RNAs
pre-mRNA: Precursor mRNA
BSJ: Back splicing junction
ciRNA: Intronic circRNA
EIciRNA: Exonic and intronic circRNA
EcircRNA: Exon circRNA
mecciRNA: Mitochondrial circRNA genomes
rt-circRNAs: Read-through circRNA
f-circRNA: Fused circRNA
snRNP: Small nuclear ribonucleoprotein
ROS: Reactive oxygen species
m^7^G: 5’ m7GpppN
RNA-seq: RNA-sequencing
ORF: Open reading frame
iORF: Infinite ORF
RFP: Ribosome footprinting
ribo-circRNA: Ribosome-bond circRNA
m^6^A-RIP: Methylated RNA immunoprecipitation sequencing
GBM: Glioblastoma
circ-protein: circRNA-derived protein
sORF: Smaller ORF
uORF: Upstream ORF
UTR: Untranslated Region
Ribo-seq: Ribosome profiling sequencing
RNC-seq: The ribosome nascent-chain complex profiling sequencing
eIF3: The eukaryotic translation initiation factor 3
eIF4G2: The eukaryotic translation initiation factor4G2
eIF4A: The eukaryotic translation initiation factor 4A
eIF4E: The eukaryotic translation initiation factor4E
eIF4B: The eukaryotic translation initiation factor4B
ITAFs: Trans-acting factors
hnRNPs: Heterogeneous nuclear ribonucleoproteins
hnRNP I: Heterogeneous nuclear ribonucleoprotein I
hnRNP Q: Heterogeneous nuclear ribonucleoprotein Q
QKI: Quaking
YTHDF3: YTH domain family protein 3
METTL3: Methyltransferase-like 3
rtEGFR: Rolling translation EGFR
OSC: Out of-frame stop codon
C-E-Cad: E-cadherin protein variant
SMO: The G protein-coupled-like receptor smoothened
PDK1: The 3-phosphoinositide-dependent protein kinase 1
TNBC: Triple-Negative Breast Cancer
GC: Gastric cancer
MAPK: Mitogen-activated protein kinases
OXPHOS: Oxidative phosphorylation
HCC: Hepatocellular carcinoma
GSK3β: Glycogen synthase kinase 3β
CC: Colon cancer
CRC: Colorectal cancer
MM: Multiple myeloma
CR: Cardiac remodeling
AD: Alzheimer’ s Disease
DMD: Duchenne Muscular Dystrophy
CAR-T: Chimeric antigen receptor T-cell
rAAV: Recombinant adeno-associated virus
